# *trans*-Chloridobis­(ethane-1,2-di­amine-κ^2^ *N*,*N*′)(thio­cyanato-κ*N*)cobalt(III) diammine­tetra­kis­(thio­cyanato-κ*N*)cromate(III)

**DOI:** 10.1107/S1600536814003869

**Published:** 2014-02-26

**Authors:** Julia A. Rusanova, Valentyna V. Semenaka, Roman I. Zubatyuk

**Affiliations:** a Taras Shevchenko National University, Department of Inorganic Chemistry, Volodymyrska str. 64/13, 01601 Kyiv, Ukraine; b Institute for Scintillation Materials, "Institute for Single Crystals", National Academy of Sciences of Ukraine, Lenina ave. 60, Kharkov 61001, Ukraine

## Abstract

The title ionic complex [CoCl(NCS)(C_2_H_8_N_2_)_2_][Cr(NCS)_4_(NH_3_)_2_], which crystallizes as a non-merohedral twin, is build up of a complex cation [CoCl(NCS)(en)_2_]^+^ (en is ethane-1,2-di­amine) and the Reinecke’s salt anion [Cr(NCS)_4_(NH_3_)_2_]^−^ as complex counter-ion. A network of N—H⋯S and N—H⋯Cl hydrogen bonds, as well as short S⋯S contacts [3.538 (2) and 3.489 (3) Å], between the NCS groups of the complex anions link the mol­ecules into a three-dimentional supra­molecular network. Intensity statistic indicated twinning by non-mero­hedry with refined weighs of twin components are 0.5662:0.4338.

## Related literature   

For background to the ammonium salt route for direct synthesis of coordination compounds, see: Kovbasyuk *et al.* (1997 ▶); Pryma *et al.* (2003 ▶); Buvaylo *et al.* (2005 ▶). For the salt route for direct synthesis of coordination compounds, see: Vassilyeva *et al.* (1997 ▶); Makhankova *et al.* (2002 ▶). For direct synthesis of heterometallic complexes with ethyl­enedi­amine, see: Nesterova (Pryma) *et al.* (2004 ▶); Nesterova *et al.* (2005 ▶, 2008 ▶). For the application of Reinecke’s salt in the direct synthesis of heterometallic complexes, see: Nikitina *et al.* (2008 ▶, 2009 ▶). For the structures of related complexes, see: Schubert *et al.* (1981 ▶); Tang *et al.* (1993 ▶); Foust & Janickis (1980 ▶); Anbalagan *et al.* (2009 ▶). 
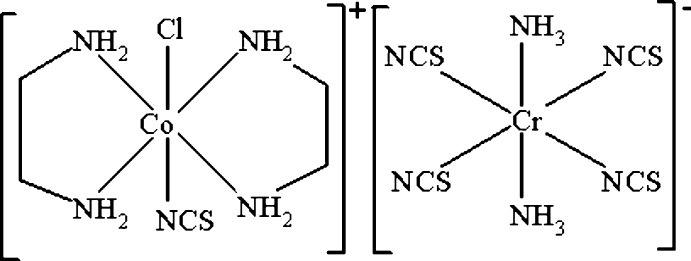


## Experimental   

### 

#### Crystal data  

[CoCl(NCS)(C_2_H_8_N_2_)_2_][Cr(NCS)_4_(NH_3_)_2_]
*M* *_r_* = 591.05
Triclinic, 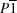

*a* = 8.8290 (15) Å
*b* = 10.745 (3) Å
*c* = 13.275 (3) Å
α = 106.98 (2)°
β = 93.131 (17)°
γ = 90.646 (17)°
*V* = 1202.1 (5) Å^3^
*Z* = 2
Mo *K*α radiation
μ = 1.71 mm^−1^
*T* = 293 K
0.27 × 0.24 × 0.08 mm

#### Data collection  

Oxford Diffraction Xcalibur Sapphire3 diffractometer
Absorption correction: multi-scan (*CrysAlis PRO*; Oxford Diffraction, 2010 ▶) *T*_min_ = 0.855, *T*_max_ = 0.883
8238 measured reflections
8238 independent reflections
6185 reflections with *I* > 2σ(*I*)

#### Refinement  

*R*[*F* ^2^ > 2σ(*F* ^2^)] = 0.052
*wR*(*F* ^2^) = 0.141
*S* = 1.03
8238 reflections
256 parameters
H-atom parameters constrained
Δρ_max_ = 1.19 e Å^−3^
Δρ_min_ = −0.72 e Å^−3^

### 

Data collection: *CrysAlis PRO* (Oxford Diffraction, 2010 ▶); cell refinement: *CrysAlis PRO*; data reduction: *CrysAlis PRO*; program(s) used to solve structure: *SHELXTL* (Sheldrick, 2008 ▶); program(s) used to refine structure: *SHELXTL*; molecular graphics: *SHELXTL*; software used to prepare material for publication: *publCIF* (Westrip, 2010 ▶).

## Supplementary Material

Crystal structure: contains datablock(s) I. DOI: 10.1107/S1600536814003869/br2236sup1.cif

Structure factors: contains datablock(s) I. DOI: 10.1107/S1600536814003869/br2236Isup2.hkl

CCDC reference:

Additional supporting information:  crystallographic information; 3D view; checkCIF report

## Figures and Tables

**Table 1:** Hydrogen-bond geometry (Å, °)

*D*—H⋯*A*	*D*—H	H⋯*A*	*D*⋯*A*	*D*—H⋯*A*
N1—H1*A*⋯S3^iii^	0.97	2.60	3.485 (5)	152
N1—H1*B*⋯S5^iii^	0.97	2.70	3.598 (5)	154
N2—H2*A*⋯S3	0.97	2.54	3.473 (4)	163
N2—H2*B*⋯S4^iv^	0.97	2.54	3.411 (4)	150
N4—H4*A*⋯Cl1^v^	0.97	2.59	3.398 (4)	141
N10—H10*B*⋯S1^iv^	0.89	2.81	3.696 (6)	171
N11—H11*C*⋯S5^vi^	0.89	2.70	3.578 (5)	168

Symmetry codes: (iii) 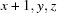; (iv) 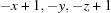; (v) 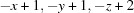; (vi) 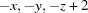.

**Fig. 1.**
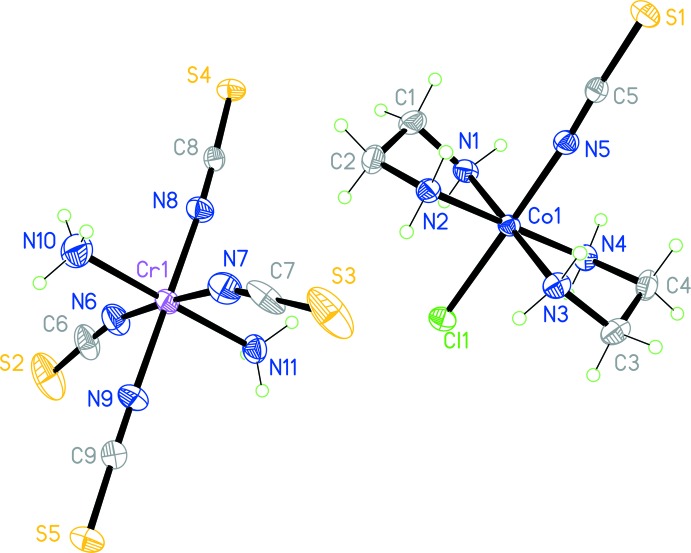
Crystal structure of the complex, showing the atom numbering, with 30% probability displacement ellipsoids

**Fig. 2.**
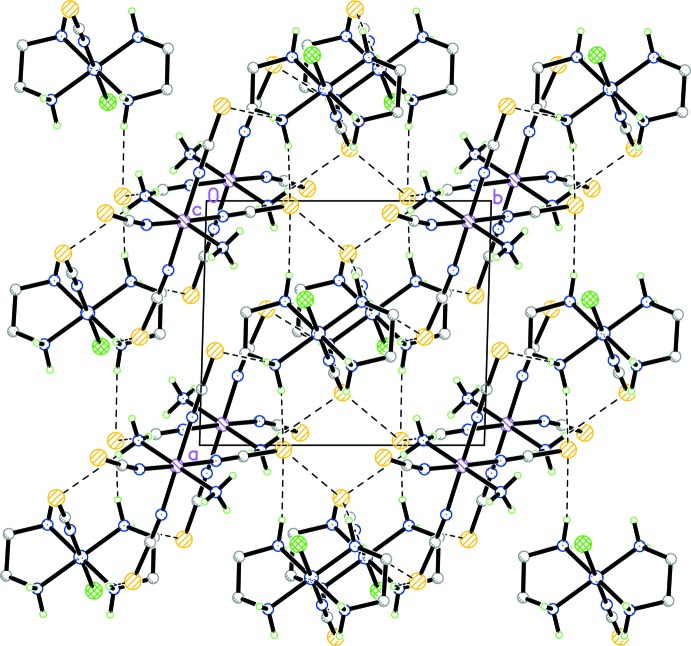
The crystal packing of the title compound. Hydrogen bonds are shown as dashed lines.

## supplementary crystallographic information

### 2.1. Synthesis and crystallization

Cobalt powder (0.074 g, 1.25 mmol), NH_4_[Cr(NCS)_4_(NH_3_)_2_]·H_2_O (0.443 g, 1.25 mmol), en·2HCl (0.166 g, 1.25 mmol) and methanol (20 ml) were heated in air to 323–333 K and stirred magnetically during 7 h. The resulting blue solution was slowly evaporated at room temperature until light-brown crystals suitable for crystallographic study were formed. The crystals were filtered off, washed with dry PriOH and finally dried *in vacuo* at room temperature. Yield: 0.12 g, 17.1%.

### 2.2. Refinement

Crystal data, data collection and structure refinement details are summarized in Table 1. All of the hydrogen atoms were positioned geometricaly and refined using a riding model approximation with *U*_iso_ = 1.2 or 1.5 *U*_eq_ of the carrier atom. A rotating model was used for NH_3_ and CH_3_ groups. Intensity statistic indicated a nonmerohedral twinning with refined weights of twin components are 0.5662:0.4338.

### 3. Results and discussion

In order to continue our research on direct synthesis of coordination compounds (Kovbasyuk *et al.*, 1997; Pryma *et al.*, 2003; Buvaylo *et al.*, 2005; Vassilyeva *et al.*, 1997; Makhankova *et al.*, 2002; Nesterova (Pryma) *et al.*, 2004; Nesterova *et al.*, 2005, 2008; Nikitina *et al.*, 2008, 2009) in this paper we present a novel Co/Cr heterometallic ionic complex which has been synthesized using zerovalent cobalt, Reinecke's salt and non-aqueous solution of ethyl­enedi­amine as a starting materials.

As it shown on Fig.1 Co atom in complex cation is in distorted square bypiramidal coordination enviroment with one NCS group and chlorine atom at the axial positions and four N atoms from two ethyl­enedi­amine molecules in equatorial plane. The Cr centers are in the similar to Co coordination enviroment and coordinated to six N atoms - four NCS-groups in equatorial position and two NH_3_ molecules in axial position. The bond distances and angles in the title molecule agree well with the corresponding bond distances and angles reported in closely related compounds (Schubert *et al.*,1981, Tang *et al.*, 1993, Foust *et al.*, 1980, Anbalagan *et al.*, 2009, Nikitina *et al.*, 2008, 2009). There are short inter­anionic S···S contacts between NCS-groups of the complex anions with the distances 3.538 (1) (S5···S5) and 3.489 (1) Å (S2···S2) whereas sum of standard Van-der-Vaals radius of the sulfur atom is 3.68 Å. Two NCS-groups of the ligand which involve S2 and S3 atoms show relatively large thermal displacements (U_eq_ is 0.1063 (9) Å^2^ and 0.0984 (8) Å^2^, resp.). Also these NCS-groups show notably non-linear Cr–N–C bond angles (166.2 (5)° and 163.2 (5)°).This might be caused by inter­molecular contacts involving S2 and S3. S···S contacts as well as a network of hydrogen bonds link the molecule into threedimentional supra­molecular network. The crystal packing of the title compound is presented on Fig 2.

### Crystal data

**Table:**

[CoCl(NCS)(C_2_H_8_N_2_)_2_][Cr(NCS)_4_(NH_3_)_2_]	*Z* = 2
*M**_r_* = 591.05	*F*(000) = 602
Triclinic, *P*1	*D*_x_ = 1.633 Mg m^−^^3^
Hall symbol: -P 1	Mo *K*α radiation, λ = 0.7107 Å
*a* = 8.8290 (15) Å	Cell parameters from 3528 reflections
*b* = 10.745 (3) Å	θ = 3.1–27.3°
*c* = 13.275 (3) Å	µ = 1.71 mm^−^^1^
α = 106.98 (2)°	*T* = 293 K
β = 93.131 (17)°	Block, light brown
γ = 90.646 (17)°	0.27 × 0.24 × 0.08 mm
*V* = 1202.1 (5) Å^3^	

### Data collection

**Table:**

Oxford Diffraction Xcalibur Sapphire3 diffractometer	8238 measured reflections
Radiation source: Enhance (Mo) X-ray Source	8238 independent reflections
Graphite monochromator	6185 reflections with *I* > 2σ(*I*)
Detector resolution: 16.1827 pixels mm^-1^	θ_max_ = 28.6°, θ_min_ = 2.9°
ω scans	*h* = −11→11
Absorption correction: multi-scan (*CrysAlis PRO*; Oxford Diffraction, 2010)	*k* = −13→13
*T*_min_ = 0.855, *T*_max_ = 0.883	*l* = −17→17

### Refinement

**Table:**

Refinement on *F*^2^	Primary atom site location: structure-invariant direct methods
Least-squares matrix: full	Hydrogen site location: inferred from neighbouring sites
*R*[*F*^2^ > 2σ(*F*^2^)] = 0.052	H-atom parameters constrained
*wR*(*F*^2^) = 0.141	*w* = 1/[σ^2^(*F*_o_^2^) + (0.080*P*)^2^ + 0.5674*P*] where *P* = (*F*_o_^2^ + 2*F*_c_^2^)/3
*S* = 1.03	(Δ/σ)_max_ = 0.001
8238 reflections	Δρ_max_ = 1.19 e Å^−^^3^
256 parameters	Δρ_min_ = −0.72 e Å^−^^3^
0 restraints	

### Special details

**Table:**

Geometry. All e.s.d.'s (except the e.s.d. in the dihedral angle between two l.s. planes) are estimated using the full covariance matrix. The cell e.s.d.'s are taken into account individually in the estimation of e.s.d.'s in distances, angles and torsion angles; correlations between e.s.d.'s in cell parameters are only used when they are defined by crystal symmetry. An approximate (isotropic) treatment of cell e.s.d.'s is used for estimating e.s.d.'s involving l.s. planes.

### Fractional atomic coordinates and isotropic or equivalent isotropic displacement parameters (Å^2^)

**Table:**

	*x*	*y*	*z*	*U*_iso_*/*U*_eq_	
Co1	0.54186 (7)	0.40606 (6)	0.75563 (4)	0.02775 (16)	
Cr1	0.08452 (9)	−0.07796 (8)	0.76057 (7)	0.0385 (2)	
Cl1	0.40444 (16)	0.35647 (13)	0.87609 (9)	0.0438 (3)	
S1	0.78440 (16)	0.49754 (14)	0.48138 (10)	0.0452 (3)	
S2	0.0503 (3)	−0.3524 (2)	0.9791 (2)	0.1063 (9)	
S3	0.0184 (2)	0.2947 (2)	0.6471 (2)	0.0984 (8)	
S4	0.55652 (16)	−0.22096 (14)	0.61991 (10)	0.0442 (3)	
S5	−0.38612 (16)	0.04575 (16)	0.91039 (11)	0.0518 (4)	
N1	0.6708 (5)	0.2569 (4)	0.7487 (3)	0.0370 (9)	
H1A	0.7764	0.2817	0.7471	0.044*	
H1B	0.6599	0.2272	0.8104	0.044*	
N2	0.4114 (4)	0.2859 (4)	0.6452 (3)	0.0343 (9)	
H2A	0.3061	0.2971	0.6623	0.041*	
H2B	0.4225	0.3036	0.5783	0.041*	
N3	0.4130 (5)	0.5558 (4)	0.7636 (3)	0.0371 (9)	
H3A	0.4180	0.5815	0.6996	0.045*	
H3B	0.3084	0.5323	0.7701	0.045*	
N4	0.6745 (5)	0.5263 (4)	0.8661 (3)	0.0367 (9)	
H4A	0.6582	0.5137	0.9342	0.044*	
H4B	0.7801	0.5104	0.8513	0.044*	
N5	0.6529 (5)	0.4460 (4)	0.6499 (3)	0.0362 (9)	
N6	0.0981 (6)	−0.2081 (5)	0.8404 (4)	0.0603 (14)	
N7	0.0637 (6)	0.0593 (5)	0.6873 (4)	0.0561 (13)	
N8	0.2839 (5)	−0.1270 (4)	0.6994 (4)	0.0461 (11)	
N9	−0.1149 (5)	−0.0282 (5)	0.8214 (4)	0.0515 (12)	
N10	−0.0200 (6)	−0.2146 (5)	0.6325 (4)	0.0695 (15)	
H10A	−0.1087	−0.2397	0.6501	0.104*	
H10B	0.0388	−0.2832	0.6125	0.104*	
H10C	−0.0353	−0.1801	0.5796	0.104*	
N11	0.1914 (5)	0.0620 (4)	0.8881 (3)	0.0472 (11)	
H11A	0.1242	0.1198	0.9190	0.071*	
H11B	0.2652	0.1023	0.8656	0.071*	
H11C	0.2306	0.0238	0.9344	0.071*	
C1	0.6231 (7)	0.1510 (5)	0.6512 (4)	0.0502 (14)	
H1C	0.6687	0.1659	0.5908	0.060*	
H1D	0.6558	0.0677	0.6578	0.060*	
C2	0.4560 (7)	0.1507 (5)	0.6370 (4)	0.0482 (13)	
H2C	0.4101	0.1210	0.6910	0.058*	
H2D	0.4220	0.0926	0.5685	0.058*	
C3	0.4661 (6)	0.6663 (5)	0.8567 (4)	0.0444 (13)	
H3C	0.4223	0.6579	0.9200	0.053*	
H3D	0.4360	0.7484	0.8463	0.053*	
C4	0.6367 (7)	0.6613 (5)	0.8674 (4)	0.0459 (13)	
H4C	0.6814	0.6831	0.8093	0.055*	
H4D	0.6757	0.7230	0.9330	0.055*	
C5	0.7041 (5)	0.4652 (4)	0.5783 (4)	0.0323 (10)	
C6	0.0819 (7)	−0.2700 (6)	0.8967 (6)	0.0664 (19)	
C7	0.0454 (6)	0.1544 (7)	0.6701 (5)	0.0591 (17)	
C8	0.3977 (5)	−0.1639 (4)	0.6657 (4)	0.0334 (10)	
C9	−0.2279 (6)	0.0020 (5)	0.8567 (4)	0.0374 (11)	

### Atomic displacement parameters (Å^2^)

**Table:**

	*U*^11^	*U*^22^	*U*^33^	*U*^12^	*U*^13^	*U*^23^
Co1	0.0309 (3)	0.0296 (3)	0.0247 (3)	0.0017 (2)	0.0031 (3)	0.0106 (3)
Cr1	0.0263 (4)	0.0419 (5)	0.0462 (5)	0.0008 (3)	0.0036 (4)	0.0108 (4)
Cl1	0.0507 (8)	0.0489 (7)	0.0341 (7)	−0.0010 (6)	0.0105 (6)	0.0143 (6)
S1	0.0434 (8)	0.0541 (8)	0.0429 (7)	−0.0030 (6)	0.0120 (6)	0.0201 (6)
S2	0.0773 (14)	0.1127 (18)	0.167 (2)	−0.0156 (12)	−0.0050 (15)	0.1033 (18)
S3	0.0516 (11)	0.1282 (18)	0.163 (2)	0.0142 (11)	0.0175 (12)	0.1146 (18)
S4	0.0379 (7)	0.0539 (8)	0.0404 (7)	0.0099 (6)	0.0089 (6)	0.0116 (6)
S5	0.0381 (8)	0.0682 (10)	0.0514 (8)	0.0099 (7)	0.0136 (6)	0.0190 (7)
N1	0.040 (2)	0.037 (2)	0.040 (2)	0.0086 (18)	0.0094 (19)	0.0181 (19)
N2	0.034 (2)	0.037 (2)	0.030 (2)	−0.0024 (17)	0.0018 (17)	0.0074 (17)
N3	0.043 (2)	0.038 (2)	0.032 (2)	0.0060 (18)	0.0011 (19)	0.0116 (18)
N4	0.040 (2)	0.038 (2)	0.033 (2)	−0.0029 (18)	−0.0041 (18)	0.0126 (17)
N5	0.042 (2)	0.035 (2)	0.033 (2)	0.0034 (18)	0.0054 (19)	0.0111 (18)
N6	0.051 (3)	0.056 (3)	0.083 (4)	0.000 (2)	0.013 (3)	0.033 (3)
N7	0.047 (3)	0.068 (3)	0.057 (3)	0.005 (3)	0.003 (2)	0.024 (3)
N8	0.033 (2)	0.050 (3)	0.055 (3)	0.005 (2)	0.008 (2)	0.014 (2)
N9	0.034 (3)	0.062 (3)	0.059 (3)	0.004 (2)	0.013 (2)	0.016 (2)
N10	0.049 (3)	0.073 (4)	0.073 (4)	−0.004 (3)	−0.002 (3)	0.002 (3)
N11	0.041 (3)	0.049 (3)	0.048 (3)	−0.010 (2)	−0.003 (2)	0.012 (2)
C1	0.068 (4)	0.032 (3)	0.051 (3)	0.011 (3)	0.021 (3)	0.009 (2)
C2	0.065 (4)	0.034 (3)	0.042 (3)	−0.011 (3)	0.004 (3)	0.007 (2)
C3	0.060 (4)	0.037 (3)	0.036 (3)	0.012 (2)	0.004 (2)	0.008 (2)
C4	0.064 (4)	0.031 (3)	0.041 (3)	−0.011 (2)	−0.003 (3)	0.008 (2)
C5	0.032 (3)	0.030 (2)	0.033 (3)	−0.0010 (19)	−0.001 (2)	0.007 (2)
C6	0.035 (3)	0.049 (4)	0.122 (6)	0.001 (3)	0.004 (4)	0.038 (4)
C7	0.029 (3)	0.105 (5)	0.063 (4)	0.003 (3)	0.008 (3)	0.053 (4)
C8	0.034 (3)	0.030 (2)	0.039 (3)	−0.001 (2)	0.000 (2)	0.014 (2)
C9	0.038 (3)	0.039 (3)	0.039 (3)	0.000 (2)	−0.003 (2)	0.017 (2)

### Geometric parameters (Å, º)

**Table:**

Co1—Cl1	2.2378 (14)	N3—H3B	0.9700
Co1—N1	1.960 (4)	N3—C3	1.492 (6)
Co1—N2	1.954 (4)	N4—H4A	0.9700
Co1—N3	1.962 (4)	N4—H4B	0.9700
Co1—N4	1.965 (4)	N4—C4	1.488 (6)
Co1—N5	1.900 (4)	N5—C5	1.144 (6)
Cr1—N6	1.987 (5)	N6—C6	1.149 (8)
Cr1—N7	1.995 (5)	N7—C7	1.122 (8)
Cr1—N8	1.990 (4)	N8—C8	1.150 (6)
Cr1—N9	1.990 (5)	N9—C9	1.135 (6)
Cr1—N10	2.058 (5)	N10—H10A	0.8900
Cr1—N11	2.080 (4)	N10—H10B	0.8900
S1—C5	1.623 (5)	N10—H10C	0.8900
S2—C6	1.629 (7)	N11—H11A	0.8900
S3—C7	1.640 (7)	N11—H11B	0.8900
S4—C8	1.615 (5)	N11—H11C	0.8900
S5—C9	1.616 (5)	C1—H1C	0.9700
S2—S2^i^	3.489 (3)	C1—H1D	0.9700
S5—S5^ii^	3.538 (2)	C1—C2	1.477 (8)
N1—H1A	0.9700	C2—H2C	0.9700
N1—H1B	0.9700	C2—H2D	0.9700
N1—C1	1.489 (7)	C3—H3C	0.9700
N2—H2A	0.9700	C3—H3D	0.9700
N2—H2B	0.9700	C3—C4	1.508 (8)
N2—C2	1.485 (6)	C4—H4C	0.9700
N3—H3A	0.9700	C4—H4D	0.9700
			
N1—Co1—Cl1	90.78 (12)	H4A—N4—H4B	108.5
N1—Co1—N3	179.61 (17)	C4—N4—Co1	107.7 (3)
N1—Co1—N4	93.44 (17)	C4—N4—H4A	110.2
N2—Co1—Cl1	88.77 (12)	C4—N4—H4B	110.2
N2—Co1—N1	86.23 (17)	C5—N5—Co1	171.6 (4)
N2—Co1—N3	94.07 (17)	C6—N6—Cr1	166.2 (5)
N2—Co1—N4	179.54 (17)	C7—N7—Cr1	163.2 (5)
N3—Co1—Cl1	88.98 (13)	C8—N8—Cr1	175.0 (4)
N3—Co1—N4	86.27 (17)	C9—N9—Cr1	179.0 (5)
N4—Co1—Cl1	91.54 (12)	Cr1—N10—H10A	109.5
N5—Co1—Cl1	178.03 (13)	Cr1—N10—H10B	109.5
N5—Co1—N1	89.78 (17)	Cr1—N10—H10C	109.5
N5—Co1—N2	89.38 (17)	H10A—N10—H10B	109.5
N5—Co1—N3	90.47 (17)	H10A—N10—H10C	109.5
N5—Co1—N4	90.31 (17)	H10B—N10—H10C	109.5
N6—Cr1—N7	176.5 (2)	Cr1—N11—H11A	109.5
N6—Cr1—N8	92.1 (2)	Cr1—N11—H11B	109.5
N6—Cr1—N9	88.1 (2)	Cr1—N11—H11C	109.5
N6—Cr1—N10	90.7 (2)	H11A—N11—H11B	109.5
N6—Cr1—N11	90.3 (2)	H11A—N11—H11C	109.5
N7—Cr1—N10	91.1 (2)	H11B—N11—H11C	109.5
N7—Cr1—N11	87.9 (2)	N1—C1—H1C	110.1
N8—Cr1—N7	90.8 (2)	N1—C1—H1D	110.1
N8—Cr1—N9	179.7 (2)	H1C—C1—H1D	108.4
N8—Cr1—N10	89.1 (2)	C2—C1—N1	108.0 (4)
N8—Cr1—N11	90.47 (19)	C2—C1—H1C	110.1
N9—Cr1—N7	88.9 (2)	C2—C1—H1D	110.1
N9—Cr1—N10	90.9 (2)	N2—C2—H2C	110.2
N9—Cr1—N11	89.51 (19)	N2—C2—H2D	110.2
N10—Cr1—N11	179.0 (2)	C1—C2—N2	107.6 (4)
Co1—N1—H1A	110.1	C1—C2—H2C	110.2
Co1—N1—H1B	110.1	C1—C2—H2D	110.2
H1A—N1—H1B	108.4	H2C—C2—H2D	108.5
C1—N1—Co1	108.2 (3)	N3—C3—H3C	110.3
C1—N1—H1A	110.1	N3—C3—H3D	110.3
C1—N1—H1B	110.1	N3—C3—C4	107.1 (4)
Co1—N2—H2A	109.9	H3C—C3—H3D	108.5
Co1—N2—H2B	109.9	C4—C3—H3C	110.3
H2A—N2—H2B	108.3	C4—C3—H3D	110.3
C2—N2—Co1	108.7 (3)	N4—C4—C3	107.1 (4)
C2—N2—H2A	109.9	N4—C4—H4C	110.3
C2—N2—H2B	109.9	N4—C4—H4D	110.3
Co1—N3—H3A	109.8	C3—C4—H4C	110.3
Co1—N3—H3B	109.8	C3—C4—H4D	110.3
H3A—N3—H3B	108.2	H4C—C4—H4D	108.5
C3—N3—Co1	109.4 (3)	N5—C5—S1	176.7 (5)
C3—N3—H3A	109.8	N6—C6—S2	176.6 (6)
C3—N3—H3B	109.8	N7—C7—S3	179.0 (7)
Co1—N4—H4A	110.2	N8—C8—S4	177.7 (5)
Co1—N4—H4B	110.2	N9—C9—S5	178.3 (5)
			
Co1—N1—C1—C2	−38.5 (5)	Co1—N4—C4—C3	42.6 (5)
Co1—N2—C2—C1	−38.6 (5)	N1—C1—C2—N2	50.6 (5)
Co1—N3—C3—C4	35.7 (5)	N3—C3—C4—N4	−51.3 (5)

Symmetry codes: (i) −*x*, −*y*−1, −*z*+2; (ii) −*x*−1, −*y*, −*z*+2.

### Hydrogen-bond geometry (Å, º)

**Table:**

*D*—H···*A*	*D*—H	H···*A*	*D*···*A*	*D*—H···*A*
N1—H1*A*···S3^iii^	0.97	2.60	3.485 (5)	152
N1—H1*B*···S5^iii^	0.97	2.70	3.598 (5)	154
N2—H2*A*···S3	0.97	2.54	3.473 (4)	163
N2—H2*B*···S4^iv^	0.97	2.54	3.411 (4)	150
N4—H4*A*···Cl1^v^	0.97	2.59	3.398 (4)	141
N10—H10*B*···S1^iv^	0.89	2.81	3.696 (6)	171
N11—H11*C*···S5^vi^	0.89	2.70	3.578 (5)	168

Symmetry codes: (iii) *x*+1, *y*, *z*; (iv) −*x*+1, −*y*, −*z*+1; (v) −*x*+1, −*y*+1, −*z*+2; (vi) −*x*, −*y*, −*z*+2.
